# Excessive Work Hours of Physicians in Training: Maladaptive Coping Strategies

**DOI:** 10.1371/journal.pmed.0040279

**Published:** 2007-09-25

**Authors:** Pashtoon Murtaza Kasi, Masoom Kassi, Talha Khawar

We would like to congratulate Kenneth R. Fernández Taylor for bringing up such an important but avoided issue in developing countries like Pakistan [1]. The growing debate regarding long working hours of postgraduate trainees has been receiving considerable attention recently [2]. This greater workload contributes to increasing stress and decreases the overall performance and the quality of life of the affected individuals [3,4].

In Pakistan, physicians, after having done a five-year medical degree (MBBS) course, are supposed to do their “internship”, or “house job” as it is often referred to. The salaries speak a sorry tale as the typical monthly salary of an intern starts from 8,000 rupees (US$129); even lower than what is mentioned by the author in El Salvador.

The author very rightly describes a typical tiring working week for an intern with little or no time for any educational activities. Some of the specialties are known for the fact that their working hours are “killing” for their residents and interns; unfortunately, some may even pride themselves on this. This inhumane approach is not often criticized by the interns working in a hospital; probably because they are too tired at the end of a day or even two or three continuous days to do so. We know of two specialties (neurosurgery and urology) in which the on call team came on Friday and left on Monday morning (72 hours straight); the reason being no other team was available to cover for them. And most of the time what an intern does is merely “clerical” work, with little satisfaction.

We, as final year medical students, tried to bring attention to this issue by documenting firstly how many hours the interns and residents worked; and secondly if these hours led to negative coping strategies or mechanisms, which might further contribute to the stress of these individuals, rather than helping them in relieving it.

We found that long working hours were indeed leading to negative coping mechanisms such as behavioral disengagement (“I’ve been giving up trying to deal with it”), substance use (“I’ve been using alcohol or drugs to make myself feel better”), denial (“I’ve been saying to myself, ‘this isn’t real’”), and venting (“I’ve been saying things to let my unpleasant feelings escape”). The frequency of different coping strategies employed by the residents in the past two weeks was determined with the Brief COPE–28 tool [5].

We also found significant levels of mild as well as morbid stress in the trainees of the hospital, with every second individual suffering from some degree of stress as well. Action indeed is needed.
